# Dissecting the reciprocal association between cytoskeletal mechanics and senescence-associated phenotypes in human dermal fibroblasts

**DOI:** 10.3389/fphys.2026.1882078

**Published:** 2026-10-20

**Authors:** Han Tang, Ruonan Qian, Ning Wang, Yu Pan, Lingling Xuan, Jing Wang, Hu Huang, Xiangnan Zhang

**Affiliations:** 1 Institute of Pharmacology & Toxicology, State Key Laboratory of Advanced Drug Delivery and Release Systems, Zhejiang Key Laboratory of Neuropsychopharmacology, College of Pharmaceutical Sciences, Zhejiang University, Hangzhou, China; 2 International Institute for Science, Proya Cosmetics Co., Ltd., Hangzhou, China; 3 Zhejiang Provincial Engineering Research Center for Innovative Small‐Molecule Drugs, Jinhua Institute of Zhejiang University, Jinhua, China

**Keywords:** cytoskeletal mechanics, extracellular matrix, F-actin organization, photoaging, senescence−associated phenotypes

## Abstract

**Introduction:**

Cellular mechanical properties play crucial roles in governing cellular functions, with their dynamic changes influenced by various factors including physiological and pathological states. Skin photoaging is closely linked to fibroblast senescence and extracellular matrix (ECM) dysfunction, yet the interplay between cellular senescence and cytoskeletal mechanics remains poorly understood. This study aimed to characterize cytoskeletal mechanical changes and explore whether maintaining F-actin cytoskeletal stability could attenuate senescent phenotypes in photoaged human dermal fibroblasts (HDFs).

**Methods:**

A model of HDFs with senescence-associated phenotypes was established by ultraviolet B (UVB) irradiation. Micromechanical properties of UVB-irradiated HDFs were evaluated using integrated cellular, molecular, and biophysical approaches, encompassing the analysis of cellular apparent modulus, cell-mediated ECM contraction, and actin cytoskeletal contractile units. Latrunculin A (LAT-A) and Jasplakinolide (JASP) were employed for targeted remodeling of the F-actin cytoskeleton to evaluate their effects on cellular senescence-associated markers.

**Results:**

UVB-irradiated HDFs with senescence-associated phenotypes exhibited reduced apparent Young’s modulus, impaired F-actin polymerization and organization, diminished myosin II regulatory light chain phosphorylation, and compromised collagen matrix contraction. Disruption of F-actin polymerization with LAT-A in young HDFs was accompanied by the appearance of cellular senescence markers, including upregulation of p21 and MMP1, downregulation of Lamin B1 and COL I. Conversely, stabilizing F-actin with JASP in UVB-exposed HDFs attenuated these changes, preserved spindle-shaped cellular morphology, and reduced the expression of senescence markers.

**Discussion:**

Our findings confirm that UVB-induced senescence-associated phenotypes in HDFs are accompanied by impaired F-actin cytoskeletal mechanics, and pharmacological modulation of F-actin partially alters multiple senescence-associated markers. These findings provide theoretical evidence and potential intervention strategies to disrupt the establishment of the UVB-induced senescent phenotype in HDFs.

## 1 Introduction

The mechanical properties of cells, as fundamental biophysical characteristics, are closely associated with the regulation of cellular proliferation, differentiation, migration, and homeostasis maintenance (Park et al., 2020; Gupta and Chaudhuri, 2022). These mechanical properties can be influenced by alterations in external forces during metabolic processes as well as by aging-related changes (Evers et al., 2021; Palmer and Jensen, 2022). The cytoskeleton—particularly filamentous actin (F-actin)—serves as the primary structural determinant of cellular mechanics, interacting with myosin to generate contractile forces that drive cell shape dynamics and migration (Blanchoin et al., 2014). A bidirectional regulatory loop exists between cellular mechanics and ECM homeostasis. Cells drive ECM remodeling by exerting traction forces and contracting the collagen matrix. In turn, ECM-derived signals engage cell surface receptors to reciprocally modulate cellular mechanical properties, forming a dynamic feedback network (Silverman et al., 2024; Hong et al., 2025).

Skin aging is a complex physiological process governed by the interplay of intrinsic genetic factors and extrinsic environmental stimuli, and is characterized by progressive structural and functional decline (Yaar, 2006; Farage et al., 2008). Human dermal fibroblasts (HDFs), the predominant functional cells in the dermis, sustain skin integrity through the synthesis and secretion of ECM components, including collagen fibers (Cole et al., 2018; Plikus et al., 2021). Additionally, HDFs exert tensile forces on the surrounding ECM (Harris et al., 1981; Baker et al., 2015), thereby generating prestress within the ECM network that contributes to the mechanical integrity of dermal tissue (Tomasek et al., 2002; Bischofs et al., 2008). Cellular senescence of fibroblasts constitutes a pivotal driver of skin aging and directly disrupts ECM homeostasis, resulting in elevated collagen degradation, diminished collagen biosynthesis, and increased skin fragility (Marcos-Garcés et al., 2014; Park, 2022). Among extrinsic stressors, ultraviolet (UV) radiation represents the principal etiological factor in photoaging. UV exposure induces fibroblast senescence via multiple signaling pathways and is associated with aberrant expression of senescence-associated molecular markers (Lee and Liu, 2020; Wyles et al., 2024). Significant progress has been made in understanding the molecular mechanisms of skin aging, fibroblast senescence, and associated changes in ECM composition. However, dynamic alterations in cellular biophysical phenotypes and the regulatory principles that govern this process remain poorly understood.

Recently, the interdisciplinary field bridging cell biology and biophysics has garnered increasing attention, with emerging studies focusing on mechanical alterations in dermal fibroblasts during skin aging. Accumulating evidence indicates reduced elasticity, weakened cytoskeletal forces in replicative senescent fibroblasts, as well as marked alterations in F-actin-associated regulators in aged fibroblasts (Rebehn et al., 2023; Boismal et al., 2024). These findings suggest that cytoskeletal mechanical dysregulation may contribute to the progression of cellular senescence. Nevertheless, it remains inconclusive whether reciprocal interactions exist between cytoskeletal mechanics and cellular senescence-associated phenotypes during photoaging. More importantly, therapeutic strategies that target fibroblast senescence by modulating cellular mechanics to restore ECM homeostasis are still in early stages of exploration. Such approaches currently lack clearly defined molecular targets and a solid theoretical foundation.

This study aims to systematically investigate the dynamic alterations of cellular mechanical phenotypes in UVB-exposed HDFs and clarify the effects of pharmacological modulation of F-actin polymerization on senescence-related markers. We first established a UVB-induced senescence-like model in HDFs. Subsequently, an integrated approach combining cell biology, molecular biology, and biophysics was employed to characterize the apparent cellular stiffness, actomyosin activity, and ECM contraction of UVB-irradiated fibroblasts. Moreover, the effects of pharmacological modulation of F-actin polymerization on fibroblast senescence-associated phenotypes were further evaluated, thereby elucidating a reciprocal association between these phenotypes and cytoskeletal mechanics.

## 2 Materials and methods

### 2.1 Cell culture

Human dermal fibroblasts (HDFs; Cat. No. PC2031. Lot no. 230131, 230821, 241227, Guangdong BioCell Biotech Co., Ltd., China) were cultured in low-glucose Dulbecco’s Modified Eagle Medium (DMEM; A18967-01, Gibco, USA) supplemented with 10% fetal bovine serum (FBS; 10099141C, Gibco) and 1% penicillin/streptomycin (15140-122, Gibco). Cells were cultured in a humidified 5% CO_2_ incubator at 37 °C, with the culture medium refreshed every day. Upon reaching approximately 90% confluence, cells were either passaged or seeded into plates. HDFs within the first 10 passages were considered young. Young HDFs were treated for 24 h with either 120 nM Latrunculin A (LAT-A; HY-16929, MedChemExpress, China) or 30 μM Blebbistatin (Bleb; HY-13813, MedChemExpress). For the LAT-A withdrawal assay, cells were recovered for 24 h in drug-free medium after LAT-A wash-off before functional measurements. UVB-exposed HDFs were pretreated with 20 nM Jasplakinolide (JASP; 102396-24-7, MedChemExpress) for 3 h prior to each irradiation. Treatment of young HDFs with JASP demonstrates that JASP exhibits no cytotoxicity in young, normal HDFs at the concentration used in this experiment. Appropriate vehicle (DMSO) controls showed no cytotoxicity in young, normal HDFs.

### 2.2 Establishment of a UVB-induced senescence-like model in HDFs

HDFs were seeded into cell culture plates and cultured for 24 h. The culture medium was replaced with phosphate-buffered saline (PBS) to cover the cell monolayer, after which the cells were exposed to UVB radiation at a dose of 150 mJ/cm^2^ (delivered over 10 min at an irradiance of 0.25 mW/cm^2^) using a calibrated UV irradiation system. The control cells (also maintained in PBS) were kept out of the light. Short-term exposure to PBS does not compromise cell viability (Muraki et al., 2006). Exposures were administered at 24-h intervals. All assays were performed 24 h after the final UV exposure. The experimental group receiving a single UV irradiation was designated as UV-1, and the group subjected to two irradiations administered at 24-h intervals was designated as UV-2 (**Figure 1A**).

**Figure 1:**
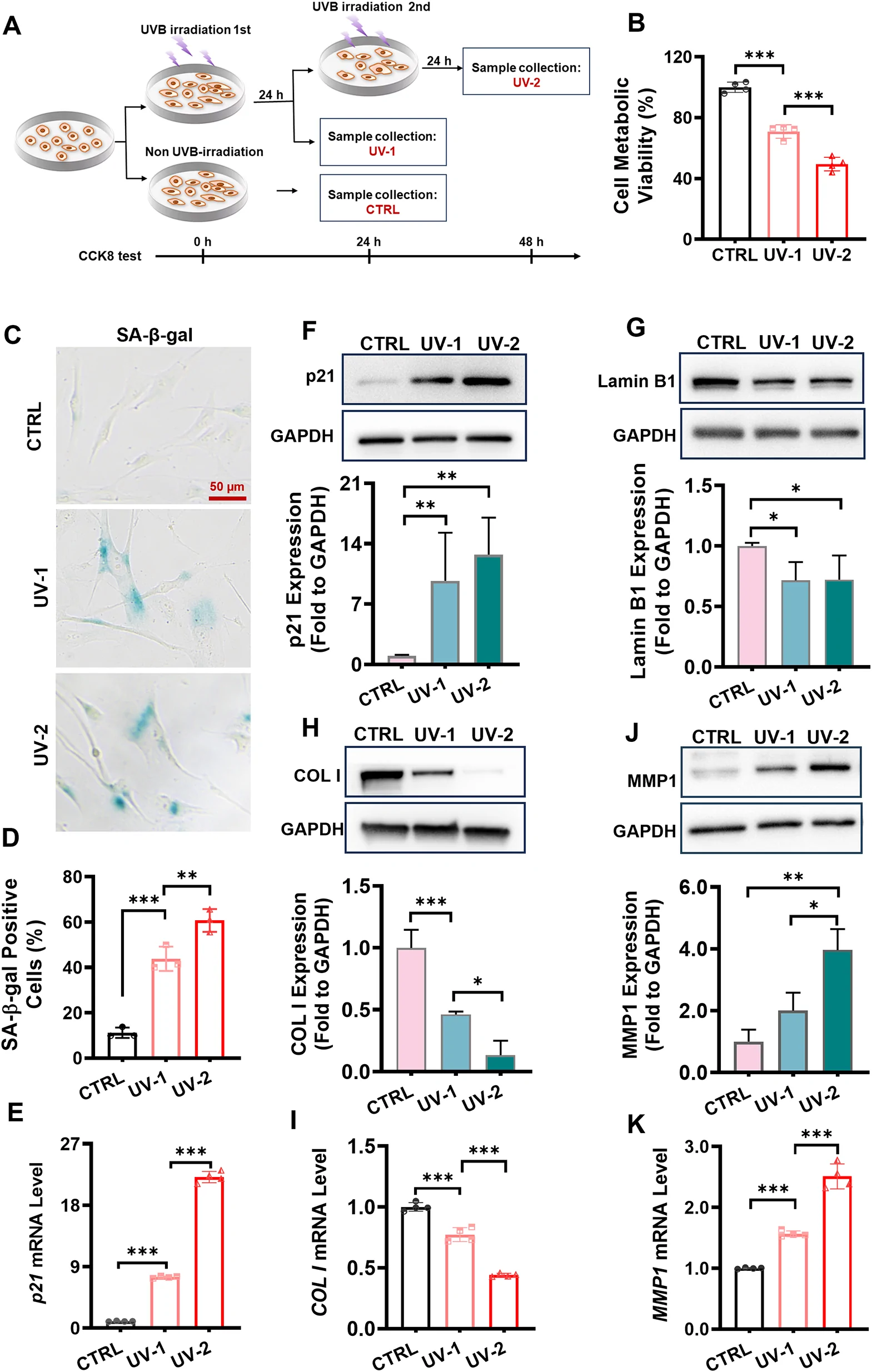
UVB irradiation induces senescence-associated phenotypes in human dermal fibroblasts (HDFs). **(A)** Schematic illustration of UVB-irradiated HDFs. **(B)** Cell metabolic activity assessed after one or two rounds of UVB irradiation. **(C)** Representative images of senescence-associated β-galactosidase (SA-β-gal) staining in HDFs following UVB irradiation. **(D)** Quantification of the percentage of SA-β-gal–positive cells after irradiation. At least 300 cells per group from three independent experiments. **(E, F)** RT-qPCR and Western blot analyses of p21 expression. **(G)** Western blot analysis and quantification of Lamin B1 protein levels. **(H, I)** RT-qPCR and Western blot analyses of COL I expression. **(J, K)** RT-qPCR and Western blot analyses of MMP1 expression. Data are presented as mean ± SD of at least three independent biological replicates (n=3). Statistical significance was determined using one-way ANOVA followed by Tukey’s *post hoc* test (two-sided). *P < 0.05, **P < 0.01, ***P < 0.001.

### 2.3 Cell metabolic activity and proliferation assays

HDFs were seeded into 24-well plates at a density of 2 × 10^4^ cells per well. Twenty-four hours after UVB irradiation, cellular metabolic activity was assessed using the CCK-8 assay kit (K1018, APExBIO, USA). The culture medium in each well was aspirated, and 300 µL of fresh medium containing CCK-8 reagent (mixed at a ratio of 10:1, v/v) was added. Plates were incubated at 37 °C in the dark for 2 h. Subsequently, 100 µL of the reaction mixture from each well was transferred to a 96-well plate, and the optical density (OD) was measured at 450 nm using a microplate reader (Spark, Tecan, Austria). Ki67 was assessed using a Ki67 cell proliferation assay kit (C2301S, Beyotime, China) according to the manufacturer’s instructions.

### 2.4 Senescence-associated β-galactosidase staining

SA-β-gal staining was performed using a β-galactosidase staining kit (C0602, Beyotime, China) according to the manufacturer’s instructions. Briefly, following completion of culture in 12-well plates, cells were fixed and incubated with the staining solution overnight at 37 °C in an incubator without CO_2_. SA-β-gal–positive cells were visualized under an inverted light microscope (Ts2R, Nikon, Japan), and the percentage of positive cells was quantified using FIJI software.

### 2.5 Cytoskeletal organization and cell morphology analysis

HDFs were seeded onto glass coverslips placed in 24-well plates at a density of 3 × 10^4^ cells per well. Cells were subjected to fluorescent staining of F-actin and nuclei to assess F-actin cytoskeletal architecture and cell morphology. Specifically, cells were then fixed with 4% paraformaldehyde for 30 min and permeabilized with 0.2% Triton X-100 for 10 min. F-actin was labeled by adding 200 µL of Alexa Fluor 568 Phalloidin (diluted 1:200 in PBS; 40734ES75, Yeasen, China) and incubating for 30 min at room temperature in the dark. After three additional 5-min PBS washes, nuclei were counterstained with 1 µg/mL DAPI (C1006, Beyotime) for 10 min. Stained samples were imaged within 24 h using an Olympus FV3000 confocal microscope (Japan). Image analysis was performed using Fiji software on maximum-intensity projections (MIPs) of Z-stack images to quantify cell spreading area, aspect ratio, circularity, and semi-quantitative F-actin intensity. FibrilTool plugin was used to analyze F-actin fibers orientation (Boudaoud et al., 2014). Following acquisition of F-actin images, anisotropy parameters were calculated for both young and senescent HDFs to compare their mechanical phenotypes. The anisotropy score was interpreted as follows: a value of 0 indicates no preferred orientation (fully isotropic), while a value of 1 represents perfect alignment (fully anisotropic). For measurement of F-actin fluorescence, single-cell regions of interest (ROIs) were manually traced along the full cytoplasmic boundary using the freehand selection tool in Fiji to obtain the mean fluorescence intensity (Mean Gray Value). Mean background fluorescence intensity was acquired from blank regions adjacent to target cells. The corrected single-cell mean fluorescence intensity of F-actin was calculated by subtracting the mean fluorescence intensity of adjacent blank regions from the measured mean fluorescence intensity of each cellular ROI. For quantitative imaging, all experimental groups were acquired with identical parameters (exposure time, laser power, gain, offset).

### 2.6 Immunofluorescence staining

HDFs were fixed with 4% paraformaldehyde for 30 min and permeabilized with 0.2% Triton X-100 for 10 min. Then, cells were rinsed three times with PBS and blocked for 30 min at room temperature with 10% goat serum (16210064, Gibco) containing 0.3 M glycine (55-40-6, BBI, China) to minimize non-specific binding. Cells were incubated overnight at 4 °C with primary antibodies diluted in PBST (PBS containing 0.1% Tween-20) containing 1% bovine serum albumin (BSA; B2064-50G, Sigma-Aldrich, USA): phospho–myosin light chain 2 (p-MLC) (1:50, 3671S, Cell Signaling Technology, USA) for myosin II activation examination. p21 (1:100, ab109520, Abcam, UK) for cell cycle arrest examination. DNase I antibody (1:500, PA5-22017, Invitrogen, USA) for G-actin examination. Subsequently, cells were incubated with fluorophore-conjugated goat anti-rabbit IgG (H+L) (1:200; ab150077, Abcam, UK) for 2 h at room temperature in the dark. γ-H2AX immunofluorescence kit (C2038S, Beyotime, China) was utilized to DNA damage examination following the manufacturer’s instructions. Finally, F-actin was stained with phalloidin and cell nuclei were stained with DAPI as needed. Immunofluorescence images were acquired within 24 h using an inverted fluorescence microscope (Ts2R, Nikon). For quantification of the F-actin/G-actin ratio, single-cell regions of interest (ROIs) were manually traced along the full cytoplasmic boundary using the freehand selection tool in Fiji for the F-actin and G-actin imaging channels, respectively. Fluorescence intensities were quantified for each cellular ROI of F-actin and G-actin, while mean fluorescence intensity was measured from blank background regions adjacent to target cells. The corrected integrated density (IntDen) was calculated by subtracting the product of the mean blank-region fluorescence intensity and the corresponding cellular ROI area from the measured fluorescence intensity of each cellular ROI. The F-actin/G-actin ratio was subsequently derived from these corrected IntDen values. For quantitative imaging, all experimental groups were acquired with identical parameters (exposure time, laser power, gain, offset).

### 2.7 Collagen contraction assay

The collagen contraction assay was performed to evaluate cellular contractility (reflecting cytoskeletal prestress) by encapsulating HDFs in rat tail type I collagen (COL I) gels according to the manufacturer’s protocol (CBA-5020, Cell Biolabs). Briefly, trypsinized cells were resuspended at a density of 5 × 10^6^ cells/mL and mixed at a 1:4 ratio with a collagen working solution (stock collagen solution, PBS, and neutralization buffer). A volume of 500 µL of the mixture was pipetted into each well of a pre-chilled 24-well plate and incubated for 30–60 min to allow gelation, followed by the addition of 1 mL culture medium per well. Gel images were captured at 0.5, 6, 24, 30, and 48 h after polymerization, with a ruler as a spatial reference. Contraction dynamics were determined by quantifying gel area changes using Fiji software.

### 2.8 Single-cell nanoindentation testing

For each experimental group, a total of 20–40 individual cells were randomly selected for mechanical characterization using an optical fiber-based interferometry nanoindenter (Optics11 Life, Pavone, Netherlands). A spherical probe with a radius of 3 μm was employed, with a cantilever spring constant of 0.017 N/m. The Displacement Control mode indentation was first used to identify a proper probe force and then the Peak Poking Control mode (max load 0.03 μN) was used for measurements. The probe was advanced toward the cell at a controlled speed of 30 μm/s until a predefined peak load of 0.03 μN was reached, and then retracted at the same speed. The apparent Young’s modulus was determined by fitting the loading segment of the force–indentation curves to the Hertz contact mechanics model according to the manufacturer’s instructions. Indentation tests were conducted at the perinuclear cytoplasmic region of cells. This region possesses sufficient thickness to minimize substrate interference and contains abundant organized F-actin, making it a promising local observation region for reflecting cytoplasmic mechanical properties modulated by F-actin rearrangement (Kihara et al., 2011; Abidine et al., 2018). Analysis was conducted using DataViewer software (v2.5.2, Optics11 Life) within the linear region of the loading curve (50%), with a goodness-of-fit threshold of R^2^ =0.99.

### 2.9 RT-qPCR

Total cellular mRNA was extracted using FreeZol reagent (R711, Vazyme, China) according to the manufacturer’s instructions. First-strand cDNA was synthesized using HiScript^®^ IV All-in-One Ultra RT SuperMix (R433, Vazyme). RT-qPCR was performed using Taq Pro Universal SYBR qPCR Master Mix (Q712, Vazyme) on an Applied Biosystems™ QuantStudio™ 3 Real-Time PCR System (Thermo Fisher Scientific, USA). All gene expression levels were normalized to the housekeeping gene glyceraldehyde-3-phosphate dehydrogenase (GAPDH), and relative expression was calculated using the 2^−ΔΔCt^ method. Primer sequences for target genes are listed in **Table 1**.

**Table 1:** Primer sequences for RT-qPCR.

Gene	Forward primer sequence (5’-3’)	Reverse primer sequence (5’-3’)
*GAPDH*	TCCAAAATCAAGTGGGGCGA	AAATGAGCCCCAGCCTTCTC
*p21*	CTCAAATCGTCCAGCGACCT	GACTCCTTGTTCCGCTGCTA
*COL I*	GAGGGCCAAGACGAAGACATC	CAGATCACGTCATCGCACAAC
*MMP1*	ATCGGGGCTTTGATGTACCC	CGATGGGCTGGACAGGATTT
*Cofilin-1 (CFL1)*	CATGGAAGCAGGACCAGTAAG	CCTGTGTCCCAACCTTGAAA
*Coronin-1C (CORO1C)*	GTCCTCCCTCTGCACAAGAC	TCGTTATGTGGGCACCAGTC
*Filamin B (FLNB)*	AGGAGGGGCTGTATGCTGTA	TTCCACCGTTAAGCCCAGAC

### 2.10 Western blot analysis

Total cellular proteins were extracted using RIPA lysis buffer (WB3100, NCM, China) supplemented with a protease and phosphatase inhibitor cocktail (P1045, Beyotime). Protein samples normalized by BCA assay were separated by SDS-PAGE and transferred onto PVDF membranes (ISEQ00010-1EA, Millipore). Membranes were blocked at room temperature for 4 h with either 5% non-fat milk (for total protein detection) or 5% BSA (for phosphoprotein detection) in TBST (Tris-buffered saline with 0.1% Tween-20). Subsequently, membranes were incubated overnight at 4 °C with primary antibodies. Following three 10-min washes with TBST (T1081, Solarbio, China), membranes were incubated with HRP-conjugated secondary antibodies at 37 °C for 1 h. Then, immunoreactive bands were visualized using enhanced chemiluminescence (ECL) substrate (ECL Star, Beyotime). Band intensities were quantified using FIJI software.

Primary antibodies used were as follows: rabbit polyclonal anti-p21 (1:1,000; A19094, ABclonal), rabbit polyclonal anti–phospho–myosin light chain 2 (p-MLC2; 1:500; 3671S, Cell Signaling Technology), mouse monoclonal anti–myosin light chain 2 (MLC2; 1:1,000; 20324, ProMab), rabbit polyclonal anti–Lamin B1 (1:10,000; 12987-1-AP, Proteintech), rabbit polyclonal anti–Collagen I (1:2,000; 14695-1-AP, Proteintech), rabbit polyclonal anti–MMP1 (1:1,000; ab137332, Abcam), rabbit polyclonal anti–α-SMA (1:20,000; 80008-1-RR, Proteintech), rabbit monoclonal anti–phospho–Cofilin (1:1000; ab283500, Abcam), rabbit polyclonal anti–Cofilin (1:1000; ab42824, Abcam), and rabbit polyclonal anti–GAPDH (1:10,000; 10494-1-AP, Proteintech). HRP-conjugated secondary antibodies included goat anti-rabbit IgG (1:10,000; SA00001-2, Proteintech) and goat anti-mouse IgG (1:10,000; SA00001-1, Proteintech).

### 2.11 Statistical analysis

All experiments were independently performed at least three times (biological replicates), and data are presented as mean ± standard deviation (SD). Statistical analyses were performed using GraphPad Prism software (version 8.0). For two-group comparisons, the unpaired Student’s t-test was applied. For multiple-group comparisons, one-way analysis of variance (ANOVA) followed by Tukey’s *post hoc* test (two-sided) was applied. Statistical significance was denoted as follows: *P < 0.05, **P < 0.01, or ***P < 0.001.

## 3 Results

### 3.1 UVB-induced senescence-associated phenotypes in HDFs

An exogenous photoaging cellular model was established by UVB irradiation (**Figure 1A**) to investigate the relationship between senescence-associated phenotypes and alterations in cellular mechanical properties. CCK-8 assays showed that a single UVB exposure significantly reduced cell metabolic activity by approximately 30% relative to the control (CTRL) group, and repeated exposure decreased metabolic activity by approximately 50% (**Figure 1B**). SA-β-gal staining showed a marked increase in the proportion of positively stained cells following UVB irradiation compared to untreated controls (**Figures 1C, D**). RT-qPCR and Western blot analyses confirmed upregulation of the cyclin-dependent kinase inhibitor p21 in irradiated HDFs (**Figures 1E, F**), along with downregulation of the nuclear envelope protein Lamin B1 (**Figure 1G**). Furthermore, UVB irradiation led to a substantial reduction in COL I expression (**Figures 1H, I**) and increase in matrix metalloproteinase 1 (MMP1) expression (**Figures 1J, K**). Together, these findings demonstrate that UVB irradiation effectively induces a senescent phenotype in HDFs. Moreover, the significantly decreased expression of Ki67 and the significantly elevated levels of γH2AX, which remained detectable at 7 days post-irradiation, to some extent confirmed the persistence of the senescent phenotype (**Supplementary Figures S1A, B**).

### 3.2 Decreased cellular mechanical properties in UVB-irradiated HDFs

To elucidate senescence-associated changes in cellular mechanical properties, we examined the apparent Young’s modulus and ECM contractility of HDFs. The results obtained from perinuclear cytoplasmic measurements showed that young HDFs exhibited a significantly higher apparent Young’s modulus (≈ 4.3 kPa) compared to UVB-irradiated HDFs with senescence-associated phenotypes (UV-1: ≈ 2.9 kPa, UV-2: ≈ 2.5 kPa) (**Figure 2A**; **Supplementary Figure S2A**). Likewise, apparent Young’s modulus measured at the peripheral cytoplasm followed a similar trend albeit with higher values (**Supplementary Figure S2B**), confirming the universal decline in apparent Young’s modulus throughout UVB-irradiated HDFs. Over a 48-h period, collagen gel area was determined at five time points (0.5, 6, 24, 30, and 48 h; **Supplementary Figure S2C**) to assess cellular ECM contraction capacity. Young HDFs exhibited substantially stronger contractility than UVB- irradiated HDFs at all time points. Representative data at 24 h revealed a collagen contraction rate of approximately 80% in young HDFs, compared to approximately 70% and 43% in the UV-1 and UV-2 groups, respectively (**Figure 2B**). Consistent with this functional decline, the expression of α-smooth muscle actin (α-SMA), a canonical marker of the fibroblast contractile phenotype (Hinz et al., 2001), was significantly downregulated in irradiated senescent cells (**Supplementary Figure S2D**), as previously reported for senescent HDFs (López-Antona et al., 2022).

**Figure 2:**
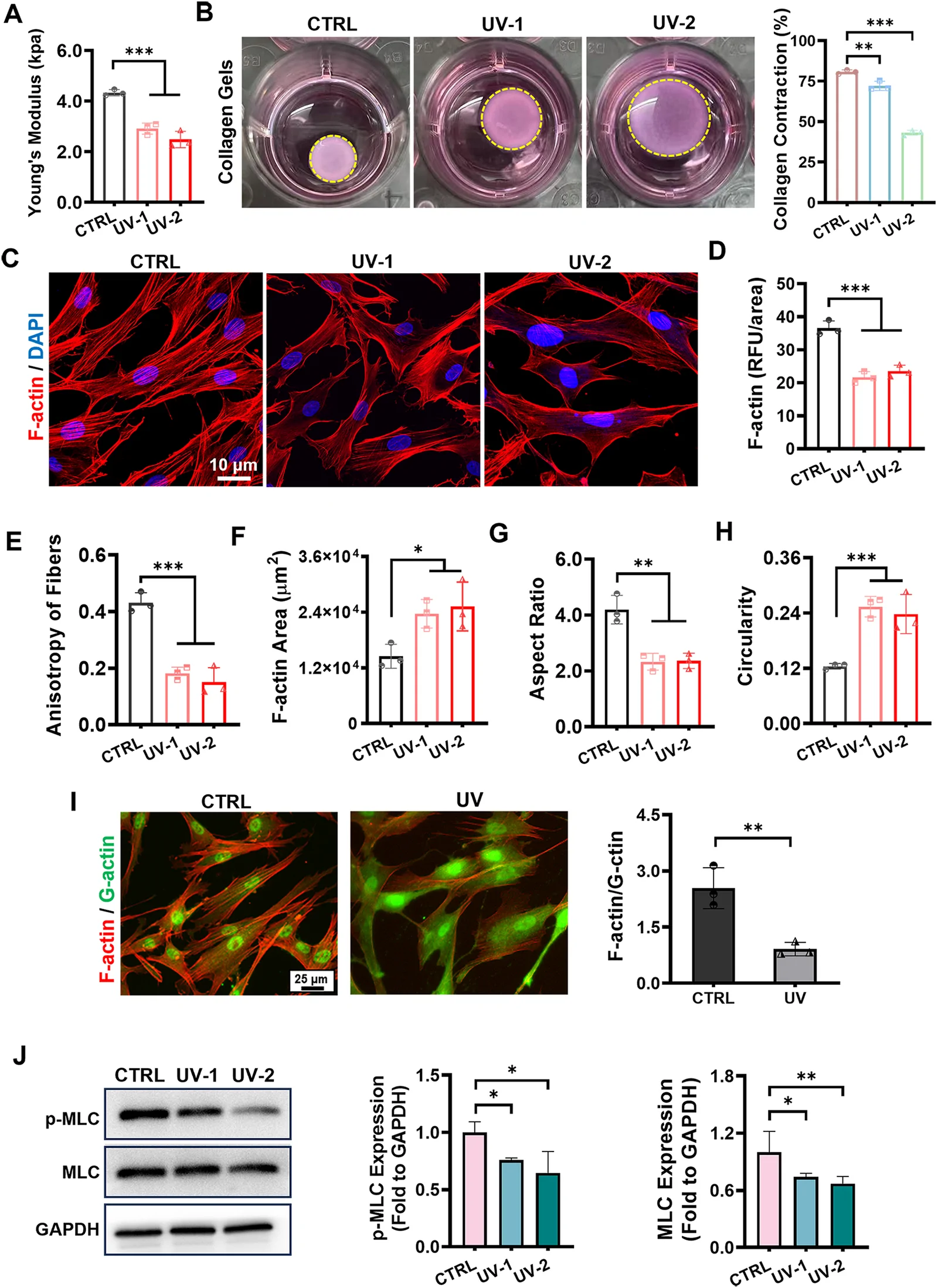
UVB irradiation impairs cellular mechanical properties. **(A)** Apparent Young’s modulus of single cells. ~60–90 cells per group from three independent experiments. One-way ANOVA followed by Tukey’s *post hoc* test (two-sided). **(B)** Representative images of collagen gels after 24 h of cell-mediated contraction, with quantification of the contraction rate. One-way ANOVA followed by Tukey’s *post hoc* test (two-sided). **(C)** Representative images of F-actin cytoskeleton (red) and nuclei (blue) in HDFs following UVB irradiations. **(D)** Quantification of F-actin assembly density. At least 50 cells per group from three independent experiments. One-way ANOVA followed by Tukey’s *post hoc* test (two-sided). **(E)** Quantification of fiber orientation anisotropy. At least 60 cells per group from three independent experiments. One-way ANOVA followed by Tukey’s *post hoc* test (two-sided). **(F)** Quantification of cell spreading area. At least 60 cells per group from three independent experiments. One-way ANOVA followed by Tukey’s *post hoc* test (two-sided). **(G)** Quantification of aspect ratio. At least 60 cells per group from three independent experiments. One-way ANOVA followed by Tukey’s *post hoc* test (two-sided). **(H)** Quantification of circularity. At least 60 cells per group from three independent experiments. One-way ANOVA followed by Tukey’s *post hoc* test (two-sided). **(I)** Immunofluorescence staining of F-actin (red) and G-actin (green) in repeatedly irradiated HDFs, with quantification of the F-actin/G-actin ratio. At least 60 cells per group from three independent experiments. Student’s t-test. **(J)** Western blot analysis and quantification of phosphorylated myosin light chain (p-MLC) and total MLC. One-way ANOVA followed by Tukey’s *post hoc* test (two-sided). Data are presented as mean ± SD from three independent biological replicates (n=3). *P < 0.05, **P < 0.01, ***P < 0.001.

The higher apparent cellular stiffness reflects, to some extent, a more robust cytoskeletal network in younger cells. Confocal imaging further illustrated that the actin cytoskeleton in UV-irradiated HDFs was sparse, particularly over the nucleus, and displayed disordered, randomly oriented arrangements (**Figures 2C–E**), in contrast to the dense, well-aligned stress fiber bundles observed in young HDFs. Morphometric analysis revealed that irradiated cells exhibited increased lateral spreading area (**Figures 2C, F**), and a shift from spindle-shaped to irregular polygonal morphology due to F-actin changes (**Figures 2C, G, H**). The reduced F-actin/G-actin ratio (**Figure 2I**), together with the unchanged total actin levels (**Supplementary Figure S2E**) in UVB-irradiated HDFs, also provides solid evidence of impaired F-actin polymerization. Furthermore, this shift in F-actin polymerization status was accompanied by elevated expression of *CFL1* (a protein governing actin severing and turnover), along with reduced levels of *CORO1C*, which mediates branched actin network organization and actin remodeling, and *FLNB*, a critical actin cross-linker and mechanical scaffold (**Supplementary Figure S2F**), as well as diminished phosphorylation of cofilin (**Supplementary Figure S2G**). Collectively, these results demonstrate that UVB affects F-actin through dysregulation of polymerization and spatial organization, rather than changes in total actin abundance. Our results further revealed that UVB-induced γH2AX was markedly upregulated at 2 h post-irradiation but declined by 24 h (**Supplementary Figures S2H, I**), whereas F-actin depolymerization and the reduction in apparent Young’s modulus only became statistically significant at 24 h (**Supplementary Figures S2H, J, K**). Persistent DNA damage lasted for up to 7 days in senescent cells, with no significant change in apparent Young’s modulus (**Supplementary Figures S1B, S2L**). These data indicate that the observed mechanical alterations are temporally uncoupled from DNA damage (Liu et al., 2022). We thus speculate that DNA damage may act as a trigger for mechanical remodeling rather than as a quantitative or sustained driver (Magalhaes et al., 2020).

Cellular contractility relies on a functional actomyosin system. Beyond F-actin, we further evaluated the expression and activation of non-muscle myosin II (NMII), a key motor protein responsible for generating cytoskeletal tension. NMII activity is controlled by phosphorylation of the myosin light chain (MLC) at Ser19 and Thr18 by serine/threonine kinases (Carmena, 2021). Using an antibody specific for MLC phosphorylated at Ser19 (p-MLC), p-MLC levels were assessed and found to be markedly reduced in UVB-irradiated HDFs (**Figure 2J**; **Supplementary Figure S2M**), consistent with impaired collagen contraction. These complementary assays consistently demonstrated that UVB-irradiated HDFs exhibited markedly reduced apparent cellular stiffness, decreased actomyosin activity, and impaired ECM contraction, indicating a comprehensive decline in cellular mechanical competence accompanying UVB-triggered senescence-associated phenotypes. Moreover, replicatively senescent HDFs (**Supplementary Figure S3A**) exhibit similar trends in mechanical phenotypic changes as UVB-irradiated HDFs, with decreased apparent Young’s modulus (**Supplementary Figure S3B**), impaired cell-mediated ECM contraction (**Supplementary Figure S3C**), altered F-actin assembly (**Supplementary Figures S3D, E**), and diminished myosin activity (**Supplementary Figures S3D, F**).

### 3.3 F-actin disruption recapitulates senescence-related mechanical and molecular alterations

F-actin is a critical determinant of cellular mechanics (Rotsch and Radmacher, 2000). Our results revealed that UVB-irradiated HDFs with senescent phenotypes exhibited reduced F-actin assembly density. To investigate whether perturbation of cytoskeletal mechanics recapitulates senescence-related mechanical and molecular alterations, young HDFs were treated with LAT-A, a potent inhibitor of F-actin polymerization. LAT-A treatment markedly decreased filamentous F-actin density (**Figure 3A**), with pronounced loss of perinuclear actin networks (nuclear outlines indicated by dashed lines). Concomitantly, the apparent Young’s modulus of LAT-A-treated cells was significantly reduced (**Figure 3B**), resembling the mechanical softening observed in UVB-irradiated HDFs. Immunofluorescence staining results showed that disruption of F-actin polymerization was accompanied by an increase in p21 expression (**Supplementary Figures S4A, B**). The Ki67-positive rate was also reduced (**Supplementary Figures S4C, D**). Western blot analysis showed significant upregulation of the senescence markers p21 and MMP1, along with downregulation of Lamin B1 (**Figure 3C**). The cellular tension-related motor protein p-MLC was also substantially decreased (**Figure 3D**). Notably, upon LAT-A withdrawal, the actin cytoskeleton recovered (**Supplementary Figure S4E**), and the expression levels of p21, Lamin B1, MMP1 and p-MLC reverted to those of control cells (**Figures 3C, D**). Furthermore, LAT-A treatment suppressed expression of α-SMA and COL I (**Supplementary Figures S4F, G**). Inhibition of myosin II activity by Bleb also altered p21 and Lamin B1 levels (**Supplementary Figure S4H**) and Ki67-positive rate (**Supplementary Figures S4C, D**). Collectively, pharmacological disruption of F-actin polymerization, was accompanied by the upregulation of multiple senescence-associated markers.

**Figure 3:**
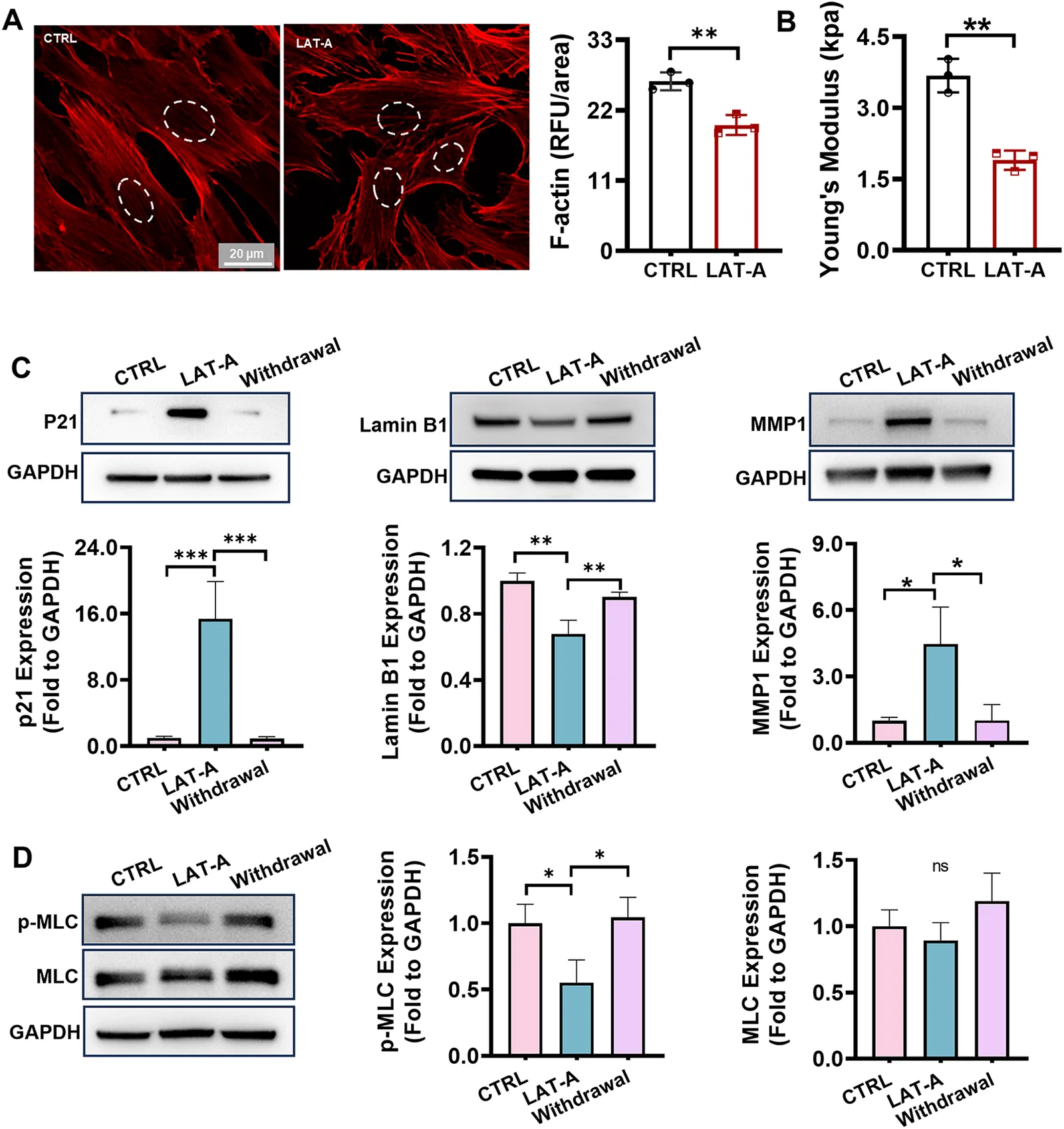
Disruption of F-actin assembly compromises cellular mechanics and promotes a senescent phenotype. **(A)** F-actin immunofluorescence staining in HDFs after 24 h of LAT-A treatment. Circles indicate nuclei. Semi-quantitative analysis of F-actin density. 60 cells per group from three independent experiments. Student’s t-test. **(B)** Measurement of single-cell apparent Young’s modulus after LAT-A treatment. ~50–80 cells per group from three independent experiments. Student’s t-test. **(C)** Western blot analysis of p21, Lamin B1, and MMP1 expression in young HDFs subjected to LAT-A treatment or after drug withdrawal. One-way ANOVA followed by Tukey’s *post hoc* test (two-sided). **(D)** Western blot analysis of p-MLC and total MLC levels in HDFs under the same conditions. One-way ANOVA followed by Tukey’s *post hoc* test (two-sided). Data are presented as mean ± SD from three independent biological replicates (n=3). *P < 0.05, **P < 0.01, ***P < 0.001.

### 3.4 Maintenance of F-actin assembly attenuates the establishment of UVB-induced senescence-associated phenotypes

To further investigate whether protecting the F-actin cytoskeleton confers anti-senescence effects, UVB-exposed HDFs were pretreated with the F-actin-stabilizing agent JASP (**Supplementary Figures S5A, B**). F-actin staining showed that JASP treatment partially preserved the elongated spindle-like morphology of UVB-irradiated HDFs, attenuating excessive lateral spreading of cells (**Figure 4A**). Meanwhile, JASP treatment protected the apparent Young’s modulus (**Figure 4B**), stabilized F-actin polymerization (**Figures 4C, D**) and preserved myosin activity (**Figures 4E, F**), indicating that JASP could mitigate UVB-impaired cellular mechanical properties in HDFs. Furthermore, the Ki67 staining suggested that JASP attenuated UVB-induced impairment of cell proliferation (**Supplementary Figures S5C, D**). Western blot analysis showed that JASP treatment downregulated the expression of p21 (**Figure 4G**). In addition, JASP treatment significantly downregulated the expression of *MMP1* (**Figure 4H**), while markedly upregulating *COL I* (**Figure 4H**). These results suggest that JASP could mitigate the impairment of cellular mechanical properties and attenuate the establishment of UVB-induced senescence-associated phenotypes in HDFs via stabilizing F-actin polymerization.

**Figure 4:**
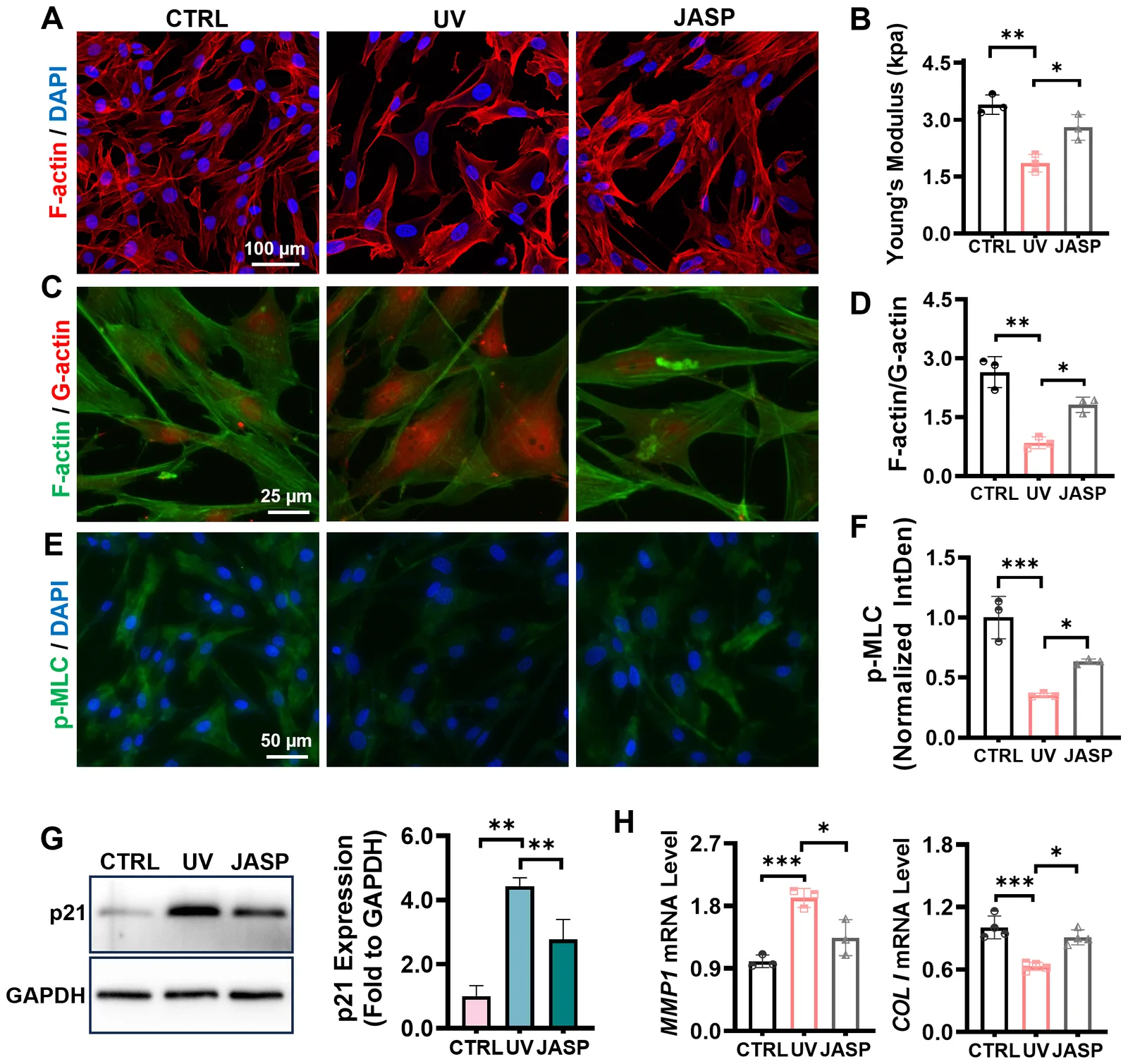
Pharmacological stabilization of F-actin attenuates senescence markers in UVB-exposed HDFs. **(A)** Representative images of F-actin **(red)** and DAPI **(blue)** staining in repeatedly irradiated HDFs with JASP pretreatment. **(B)** Apparent Young’s modulus of single cells. ~60–90 cells per group from three independent experiments. **(C, D)** Immunofluorescence staining of F-actin (green) and G-actin (red) in repeatedly irradiated HDFs, with quantification of the F-actin/G-actin ratio. 50 cells per group from three independent experiments. **(E, F)** Immunofluorescence staining and quantification of phospho–myosin light chain 2 (p-MLC) in repeatedly irradiated HDFs. 60 cells per group from three independent experiments. **(G)** Western blot analysis of p21 expression upon F-actin stabilization. **(H)** RT-qPCR analysis of *MMP1*, and *COL I* expression. Data are presented as mean ± SD from three independent biological replicates (n=3). Statistical significance was determined using one-way ANOVA followed by Tukey’s *post hoc* test (two-sided). *P < 0.05, **P < 0.01, ***P < 0.001.

## 4 Discussion

Cellular senescence is a central driver of skin aging (Chin et al., 2023). As the primary functional cells in the dermis, fibroblasts contribute to skin longevity by maintaining ECM integrity and exerting prestress on the ECM network. Their intrinsic mechanical properties not only govern their own functionality but also influence how they remodel the ECM (Lai and Wong, 2020; Tang et al., 2025). However, the interplay between cellular senescence-associated phenotypes and mechanical properties has long been underexplored in the context of skin photoaging. In this study, we established a UVB-irradiated HDF model with senescence-associated phenotypes and systematically investigated the alterations in cellular mechanical characteristics. Furthermore, we elucidated a reciprocal association between cytoskeletal mechanics and senescence-associated phenotypes.

Although a single dose of UVB irradiation was sufficient to induce senescence-associated phenotypes, repeated exposure was carried out to maintain these senescent phenotypes. The obtained results confirmed that UVB-irradiated HDFs exhibited a reduced apparent Young’s modulus. Among cytoskeletal components, F-actin is a major contributor to cellular stiffness (Rotsch and Radmacher, 2000; Chugh and Paluch, 2018). Consistent with this, UVB-irradiated HDFs displayed aberrant F-actin organization, characterized by decreased filament density and excessive spreading, particularly a marked reduction in the perinuclear actin cap. The actin cap is a highly organized, dynamic, and polarized bundle of thick actin fibers that plays a critical role in transmitting mechanical forces into the nucleus, such that its loss may impair nuclear mechanotransduction (Ma et al., 2024). Furthermore, F-actin in UV-irradiated HDFs exhibited disorganization and lost its ordered arrangement along the cellular long axis, collectively indicative of diminished actin filament tension (Burridge and Wittchen, 2013; Liu et al., 2026). Concomitantly, phosphorylation of MLC, a key indicator of NMII activity, was significantly decreased in UVB-irradiated HDFs. These coordinated disruptions in the actomyosin machinery underlie the observed impairment in collagen contraction. Since effective ECM remodeling relies on actomyosin-derived intracellular tension (Bischofs et al., 2008), UVB-irradiated fibroblasts are functionally impaired in this critical mechanical role. Collectively, our data identify reduced cellular mechanical competence as a hallmark of UVB‑irradiated HDFs with senescence-associated phenotypes (**Figure 5**). Notably, mechanical phenotypes can vary dramatically with cell type and senescence induction conditions. In contrast to our current observations of significantly reduced stiffness and contractility in HDFs, increased traction force has been documented in endothelial cells (Cheung et al., 2015), and increased stiffness in epithelial cells (Berdyyeva et al., 2005). Even within the fibroblast lineage, increased apparent cellular stiffness has been reported in aged HDFs (Schulze et al., 2010), whereas decreased stiffness was observed in replicatively senescent HDFs (Rebehn et al., 2023). These discrepancies may be attributable to intrinsic lineage-specific mechanical baselines and variations in the cellular microenvironment during the senescence process. Furthermore, methodological variations in mechanical measurement platforms, together with divergent assessment timepoints, may also contribute to the contrasting mechanical phenotypes even among cells of the same type (Nakyai et al., 2018). Collectively, the mechanical phenotypes of senescent cells are determined by the interplay of cell type, senescence induction modality, cellular microenvironment, measurement timepoints, and testing methodology. The Hertz model assumes a linearly elastic, homogeneous, isotropic, passive half-space material, which is not strictly satisfied for living contractile cells with viscoelasticity, structural heterogeneity, and intrinsic cytoskeletal pre-stress. The Young’s modulus derived from Hertz fitting herein represents an apparent, loading-rate-dependent stiffness rather than an intrinsic elastic property of HDFs (Mendová et al., 2024).

**Figure 5:**
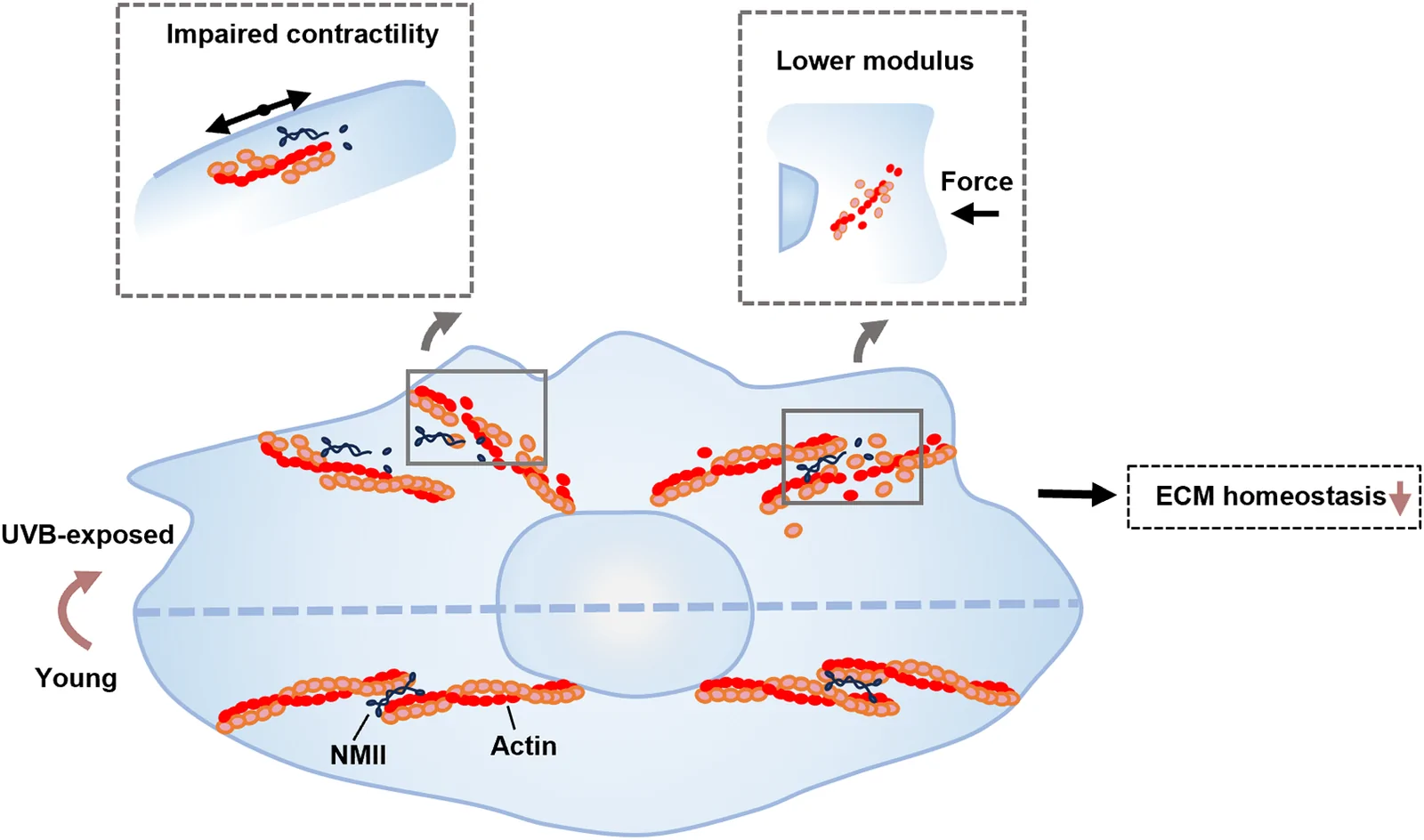
Schematic representation of cytoskeletal and mechanical function alterations in UVB-exposed HDFs with senescence-associated phenotypes. Upon UVB exposure, HDFs display sparse, disorganized, and randomly oriented F-actin filaments together with impaired NMII activity. These alterations may lead to aberrant cellular mechanical integrity and perturbed mechanosensitivity, which potentially further dysregulates ECM homeostasis. Solid lines indicate relationships supported by our experimental data, and dashed lines denote proposed mechanistic links.

Another key focus of this study was to investigate how alterations in cytoskeletal mechanics influence senescence-associated markers, with particular emphasis on the F-actin. As shown, treatment of HDFs with the actin polymerization inhibitor LAT-A disrupted F-actin filament assembly, inducing perturbed actomyosin homeostasis, loss of the perinuclear actin cap, and reduced apparent Young’s modulus. These cytoskeletal perturbations were accompanied by upregulated p21, MMP1 and downregulated Lamin B1, COL I likely due to diminished intracellular tension that disrupts nuclear structure and promotes senescence-associated phenotypic changes (Sladitschek-Martens et al., 2022). Additionally, pharmacological maintenance of F-actin polymerization by JASP effectively preserved spindle-shaped morphology, attenuated the expression of the cell cycle inhibitor (p21), and altered the expression of *COL I* and *MMP1* in UVB-exposed HDFs. This effect may stem from the resistance to F-actin depolymerization damage induced by UV oxidative stress (Zhang et al., 2019). Emerging evidence demonstrates that optimized cytoskeletal remodeling elevates chromatin accessibility to drive the expression of functional genes (Sornapudi et al., 2025). These pharmacological data support a reciprocal association between F-actin-based mechanical homeostasis and senescence-associated phenotypes. Within the dermal microenvironment, changes in ECM stiffness, fibrous architecture and biochemical composition modulate fibroblast cytoskeletal dynamics and mechanical behavior via mechanotransduction, driving the onset of cellular senescence. Senescent fibroblasts in turn exhibit impaired ECM synthetic capacity, exacerbating the deterioration of ECM structure and dermal mechanical function (Humphrey et al., 2014; Park et al., 2020; Evers et al., 2021). This creates a self-sustaining negative feedback cascade linking ECM microenvironment alteration, cytoskeletal rearrangement, cellular senescence and ECM homeostatic collapse, which collectively governs classic aging hallmarks including skin sagging, fine line formation and dermal atrophy. Elucidation of this bidirectional regulatory effect provides a fundamental framework for future research on the targeted modulation of cytoskeletal stability, restoration of the dermal microenvironment, and the rational design of anti-aging bioactive compounds and skincare strategies.

Numerous previous studies have established correlations between aberrant F-actin polymerization and cellular senescence across diverse cell types (Berdyyeva et al., 2005; Cheung et al., 2015; Rebehn et al., 2023; Liu et al., 2026). Although F-actin and mechanical properties of senescent cells vary with experimental conditions (e.g., cell types and induction methods), our study systematically characterizes the mechanical phenotype and subcellular actomyosin alterations in UVB-irradiated HDFs with senescence-associated phenotypes, beyond mere measurements of apparent Young’s modulus or senescence markers in photoaged HDFs (Lien et al., 2013; Nakyai et al., 2018).Using pharmacological modulators, we further examined the reciprocal association between F-actin stabilization and multiple senescence markers. Although Latrunculin A and Jasplakinolide can perturb actin dynamics and allow preliminary evaluation of the overall contribution of actin to cellular mechanics, this pharmacological approach has notable limitations. Beyond remodeling actin networks, these compounds interfere with cell adhesion, contractility and cell-cycle progression (Müller et al., 2013; Velle and Onishi, 2025), which renders it difficult to dissect the direct mechanical input of the actin cytoskeleton. Future experiments using mechanical stretching assays or stiffness-tunable substrates will further elucidate whether mechanical cues per se are sufficient to regulate senescence-associated phenotypes. Previous work has shown that reduced abundance of focal adhesion-targeted βPAK-interacting exchange factor (βPIX)–G protein-coupled receptor kinase-interacting protein (GIT) complex impairs integrin signaling and mediate dysregulated F-actin polymerization during cellular senescence (Shin et al., 2020). In Hutchinson–Gilford Progeria Syndrome (HGPS), aberrant F-actin polymerization drives nuclear envelope rupture and micronucleus biogenesis, which initiates innate immune signaling cascades to fuel senescence progression (Mu et al., 2020). Reduced YAP/TAZ activity in dermal fibroblasts from aged mice promotes cellular senescence. Conversely, sustained YAP function rejuvenates old cells (Sladitschek-Martens et al., 2022). Consistent with the canonical mechanotransduction cascade (Martino et al., 2018), we propose a hypothesis that UVB irradiation impairs integrin signaling and disrupts homeostasis of the RhoA-ROCK axis, which suppresses F-actin stress fiber assembly and reduces intracellular mechanical tension (Martino et al., 2018; Shin et al., 2020). These defects dampen the mechanical responsiveness of the YAP/TAZ pathway, impair nuclear mechanotransduction, and ultimately drive senescent phenotypes, alongside compromised ECM-remodeling capacity (Martino et al., 2018; Sladitschek-Martens et al., 2022). Furthermore, we also speculate that DNA damage may act as an initiating trigger for cellular mechanical remodeling. However, the mechanistic validation and the precise mechanoregulatory linkage between F-actin dysregulation and cellular senescence remain to be elucidated, which will be further addressed in future work.

## 5 Conclusion

This study employed a model of UVB-irradiated HDFs with senescence-associated phenotypes to investigate the reciprocal association between cytoskeletal mechanics and senescence-associated phenotypes. The results demonstrate that UVB‑irradiated HDFs exhibit profoundly impaired mechanical features, characterized by a reduced apparent Young’s modulus and significantly compromised capacity to contract collagen matrices. These mechanical deficits were attributed to disorganized F-actin with reduced filament density and decreased myosin II activity. These findings suggest that UVB-induced senescence-associated phenotypes are accompanied by impaired mechanical competence. Disruption of F-actin polymerization in young HDFs decreases actomyosin activity and upregulates senescence markers. Conversely, stabilization of F-actin in UVB-exposed HDFs attenuates senescence marker expression. These results indicate that pharmacological modulation of F-actin partially modulates cellular senescence-associated markers. Conservatively, strategies for maintaining actin cytoskeleton stability may provide a feasible approach for disrupting the establishment of the UVB-induced senescent phenotype.

## Data Availability

The original contributions presented in the study are included in the article/**Supplementary Material**. Further inquiries can be directed to the corresponding authors.
