# Supplementary material for: Dissecting the reciprocal association between cytoskeletal mechanics and senescence-associated phenotypes in human dermal fibroblasts
Source: Front Physiol. 2026 Oct 20;17:1882078. doi: 10.3389/fphys.2026.1882078 (PMC13727495; doi:10.3389/fphys.2026.1882078)
Supplement: Supplementary file 1 [file DataSheet1.docx]

Supplementary Material

# 1 Supplementary Figures

**Supplementary Figure 1.** Repeated UVB irradiation induced a persistent senescent phenotype. (A) Immunofluorescence staining of Ki67 and the percentage of Ki67‑positive cells. At least 450 cells per group from three independent experiments. (B) γH2AX immunofluorescence and fluorescence intensity quantification. At least 450 cells per group from three independent experiments. Data are presented as mean ± SD from three independent biological replicates (n=3). One-way ANOVA followed by Tukey’s post hoc test (two-sided) was used for multiple-group comparison. ***P* < 0.01, ****P* < 0.001.

**Supplementary Figure 2.** UVB irradiation impairs cellular mechanical properties. (A) Representative Force–indentation curves of CTRL, UV-1, UV-2. (B) Single‑cell Young's modulus at the perinuclear cytoplasm (Pn) and peripheral cytoplasm (Pp). ~60–90 cells per group from three independent experiments. Two-way ANOVA followed by Tukey’s post hoc test (two-sided). (C) Time-course quantification of collagen contraction at indicated time points. Two-way ANOVA followed by Tukey’s post hoc test (two-sided). (D)Western blot analysis and quantification of α-SMA protein expression. One-way ANOVA followed by Tukey’s post hoc test (two-sided). (E) Western blot analysis and quantification of total intracellular actin protein expression. One-way ANOVA followed by Tukey’s post hoc test (two-sided). (F) RT-qPCR analysis of mRNA expression of cytoskeletal assembly-related genes, including *Cofilin-1* (*CFL1)*, *Coronin-1C* (*CORO1C)*, and *Filamin B (FLNB)*. One-way ANOVA followed by Tukey’s post hoc test (two-sided). (G) Western blot analysis and quantification of the p-Cofilin/Cofilin ratio. One-way ANOVA followed by Tukey’s post hoc test (two-sided). (H) Representative images of γH2AX (red), F‑actin (green), and DAPI (blue) at 2 h and 24 h post‑single UV irradiation. (I) γH2AX fluorescence intensity quantification based on the staining in (H). At least 100 cells per group from three independent experiments. One-way ANOVA followed by Tukey’s post hoc test (two-sided). (J) Quantification of F-actin assembly density based on the staining in (H), At least 60 cells per group from three independent experiments. One-way ANOVA followed by Tukey’s post hoc test (two-sided). (K) Single‑cell Young's modulus measured at the perinuclear cytoplasm at 2 h and 24 h after a single UVB irradiation. ~60–90 cells per group from three independent experiments. One-way ANOVA followed by Tukey’s post hoc test (two-sided). (L) Single‑cell Young's modulus at the perinuclear cytoplasm of HDFs at 24 h and 7 d post‑repeated UVB exposure. ~60–90 cells per group from three independent experiments. One-way ANOVA followed by Tukey’s post hoc test (two-sided). (M) Immunofluorescence staining and quantification of phospho–myosin light chain 2 (p-MLC) in repeatedly irradiated HDFs. 60 cells per group from three independent experiments. Student’s t-test. Data are presented as mean ± SD from three independent biological replicates (n=3). **P* < 0.05, ***P* < 0.01, ^##^*P* < 0.01, ****P* < 0.001.

**Supplementary Figure 3.** Replicative senescence impairs the mechanical properties of HDFs. (A) Representative images of senescence-associated β-galactosidase (SA-β-gal) staining and quantitative analysis of SA‑β‑gal‑positive cells in replicatively senescent HDFs. At least 400 cells per group from three independent experiments. Yo (Young, P5-P7), Sene (Senescence, P39-40). (B) Young’s modulus of single cells. ~60–90 cells per group from three independent experiments. (C) Representative images of collagen gels after 6 h of cell-mediated contraction, with quantification of the contraction rate. (D) Representative images of p-MLC (green), F-actin (red), and nuclei (blue) in replicatively senescent HDFs. (E) Quantification of F-actin density. At least 60 cells per group from three independent experiments. (F) Quantification of p-MLC fluorescence intensity. At least 60 cells per group from three independent experiments. Student’s t-test. Data are presented as mean ± SD from three independent biological replicates (n=3). **P* < 0.05, ***P* < 0.01, ****P* < 0.001.

**Supplementary Figure 4.** Disruption of actomyosin promotes a senescent phenotype. (A) Representative immunofluorescence staining of F-actin (red) and p21(green) in repeatedly irradiated HDFs and LAT-A (24 h)-treated HDFs. (B) Correlation analysis of F‑actin and p21 based on the staining in (A). (C, D) Immunofluorescence staining of Ki67 and quantitative analysis of the percentage of Ki67‑positive cells. At least 450 cells per group from three independent experiments. One-way ANOVA followed by Tukey’s post hoc test (two-sided). (E) F-actin staining in young HDFs after treatment with Latrunculin A (LAT-A) or LAT-A withdrawal. (F, G) Western blot analysis and quantification of α-SMA, COL I protein expression following LAT-A-induced F-actin disruption. Student’s t-test. (H) Western blot analysis and quantification of Lamin B1 and p21 protein expression in HDFs treated with Blebbistatin (Bleb). Student’s t-test. Data are presented as mean ± SD from three independent biological replicates (n=3). **P* < 0.05, ***P* < 0.01, ****P* < 0.001.

**Supplementary Figure 5.** Jasplakinolide (JASP) stabilizes the F-actin and facilitates proliferation of HDFs. (A) F‑actin staining and quantitative analysis of HDFs treated with JASP. 50 cells per group from three independent experiments. Student’s t-test. (B) CCK‑8 metabolic activity assay results at 24 h following JASP treatment. Student’s t-test. (C, D) Ki67 immunofluorescence and quantitative analysis of its positive ratios in JASP-treated HDFs under repeated UVB exposure. At least 450 cells per group from three independent experiments. One-way ANOVA followed by Tukey’s post hoc test (two-sided). Data are presented as mean ± SD of at least three independent biological replicates (n=3). **P* < 0.05, ***P* < 0.01, ****P* < 0.001.
